# Chronic myelomonocytic leukemia with ring sideroblasts/*SF3B1* mutation presents with low monocyte count and resembles myelodysplastic syndromes with-RS/*SF3B1* mutation in terms of phenotype and prognosis

**DOI:** 10.3389/fonc.2024.1385987

**Published:** 2024-07-01

**Authors:** Blanca Xicoy, Helena Pomares, Mireia Morgades, Ulrich Germing, Montserrat Arnan, Mar Tormo, Laura Palomo, Elisa Orna, Matteo Della Porta, Felicitas Schulz, Marina Díaz-Beya, Ada Esteban, Antonieta Molero, Luca Lanino, Alejandro Avendaño, Francisca Hernández, Verónica Roldan, Marta Ubezio, Alberto Pineda, María Díez-Campelo, Lurdes Zamora

**Affiliations:** ^1^ Hematology Department, Institut Català d’Oncologia-Hospital Germans Trias i Pujol, Badalona; Myeloid Neoplasms Group, Josep Carreras Leukemia Research Institute-Hospital Germans Trias i Pujol, Badalona, Spain; ^2^ Hematology Department. Institut Català d’Oncologia, Hospital Duran i Reynals, Institut d’Investigació Biomèdica de Bellvitge (IDIBELL), Universitat de Barcelona, L’Hospitalet de Llobregat, Barcelona, Spain; ^3^ Department of Hematology, Oncology and Clinical Immunology, Heinrich-Heine Universitätsklinikum, Düsseldorf, Germany; ^4^ Hematology Department, Hospital Clínico Universitario de Valencia, Valencia, Spain; ^5^ Hematology Department, Hospital Universitari Vall d’Hebró, Barcelona, Spain; ^6^ Hematology Department, IRCCS Humanitas Research Hospital, Rozzano, Milan, Italy; ^7^ Hematology Department, Hospital Clínic, Barcelona, Spain; ^8^ Hematology Department, Hospital de San Pedro, Logroño, Spain; ^9^ Hematology Department, Hospital Universitario de Salamanca, Salamanca, Spain; ^10^ Hematology Department, Hospital Universitario Virgen de las Nieves, Granada, Spain; ^11^ Hematology Department, Hospital Universitario de Cruces, Barakaldo, Vizcaya, Spain

**Keywords:** CMML-RS/*SF3B1*, CMML, MDS-RS/*SF3B1*, phenotype, mutational profile, prognosis

## Abstract

**Introduction:**

Chronic myelomonocytic leukemia (CMML) and myelodysplastic syndromes (MDS) with ring sideroblasts (RS) or *SF3B1* mutation (MDS-RS/*SF3B1*) differ in many clinical features, but share others, such as anemia. RS and *SF3B1* mutation can also be found in CMML.

**Methods:**

We compared CMML with and without RS/*SF3B1* and MDS-RS/*SF3B1* considering the criteria established by the 2022 World Health Organization classification.

**Results:**

A total of 815 patients were included (CMML, n=319, CMML-RS/*SF3B1*, n=172 and MDS-RS/*SF3B1*, n=324). The percentage of RS was ≥15% in almost all CMML-RS/*SF3B1* patients (169, 98.3%) and most (125, 72.7%) showed peripheral blood monocyte counts between 0.5 and 0.9 x10^9^/L and low risk prognostic categories. CMML-RS/*SF3B1* differed significantly from classical CMML in the main clinical characteristics, whereas it resembled MDS-RS/*SF3B1*. At a molecular level, CMML and CMML-RS/*SF3B1* had a significantly higher frequency of mutations in *TET2* (mostly multi-hit) and *ASXL1* (p=0.013) and CMML had a significantly lower frequency of *DNMT3A* and *SF3B1* mutations compared to CMML/MDS-RS/*SF3B1*. Differences in the median overall survival among the three groups were statistically significant: 6.75 years (95% confidence interval [CI] 5.41-8.09) for CMML-RS/*SF3B1* vs. 3.17 years (95% CI 2.56-3.79) for CMML vs. 16.47 years (NA) for MDS-RS/*SF3B1*, p<0.001. Regarding patients with CMML and MDS, both with *SF3B1* mutation, survival did not significantly differ. CMML had a higher risk of transformation to acute myeloid leukemia (24% at 8 years, 95%CI 19%-30%).

**Discussion:**

CMML-RS/*SF3B1* mutation resembles MDS-RS/*SF3B1* in terms of phenotype and clearly differs from CMML. The presence of ≥15% RS and/or *SF3B1* in CMML is associated with a low monocyte count. *SF3B1* mutation clearly improves the prognosis of CMML.

## Introduction

Monocytosis (≥0.5 x10^9^/L and ≥10%) in peripheral blood is the hallmark of chronic myelomonocytic leukemia (CMML). A subset of myelodysplastic syndromes (MDS) presents with ring sideroblasts (RS) mostly associated with *SF3B1* (splicing factor 3B, subunit 1) mutation and are recognized as a subgroup of MDS by the 2022 World Health Organization (WHO) classification (called MDS with low blasts and mutated *SF3B1* or MDS with RS) and the International Consensus Classification of Myeloid Neoplasms and Acute Leukemias (ICC) of 2022 (called MDS with mutated *SF3B1*) (1–3).

CMML and MDS with RS/*SF3B1* mutation differ in many clinical features but share others, such as anemia. In MDS, defective erythropoiesis includes impaired early and terminal erythroid maturation, which involves the development of RS, mostly associated with the presence of *SF3B1* mutation. The clinical presentation of CMML is more heterogeneous, while anemia is often present and more frequent and severe in the myelodysplastic subtype.

Treatment with erythropoietic stimulating agents is the standard of care of symptomatic anemia in low-risk MDS and CMML (4, 5). Beyond this first line, there are limited options for the treatment of anemia in lower-risk MDS/CMML. Luspatercept is a novel activin receptor type IIB fusion ligand trap agent that has shown activity for treating anemia in patients with MDS-RS/*SF3B1* and has been approved for this indication (6–8).

RS and *SF3B1* mutation can also be found in CMML. In a study of 226 patients with CMML, 6% harbored the *SF3B1* mutation (mostly the K700E variant), with this mutation being strongly associated with the presence of ≥15% RS and mutually exclusive with mutations in other splicing genes, such as *SRSF2* and *U2AF1*. Interestingly, these other splicing mutations were also associated with the presence of RS, suggesting that this feature in CMML is not restricted to the presence of a mutant *SF3B1* clone (9). It has been suggested that *SF3B1* mutation defines a unique entity within all myeloid neoplasms, but distinctive clinical and biological features and prognosis have been observed between MDS and MDS/MPN harboring this mutation, which would argue against the idea of classifying *SF3B1*-mutant myeloid neoplasms as a single entity (10–13). On the other hand, *ASXL1* and *SRSF2* mutations are infrequent in *SF3B1* mutant CMML, which may suggest that *SF3B1* mutant CMML differs clearly from CMML without *SF3B1* mutations and this specific biological profile translates into a distinct phenotype and survival rate (12, 13).

On the other hand, monocytosis (monocyte count >0.6 x10^9^) is not uncommon in patients with MDS with low blast count (< 5%). Within the RS phenotype, 20% of patients show monocytosis and there seems to be a positive correlation between the percentage of RS and absolute monocyte count, with the overall survival (OS) being significantly shorter (14). Moreover, the absolute monocyte count at diagnosis may affect the prognosis in MDS independently of the Revised International Prognostic Score System (IPSS-R) risk score, and monocytopenia (<0.2 x10^9^/L) may be associated with a higher risk of acute myeloid leukemia (AML) transformation (15).

We aimed to describe the clinical characteristics and prognosis of CMML patients with *SF3B1* and/or ≥15% RS (CMML-RS/*SF3B1*) and compare them with CMML without this feature and with MDS-RS/*SF3B1*. We hypothesize that a proportion of RS ≥15% (or less but with the presence of *SF3B1* mutation) in the bone marrow of patients with CMML might define a subset of patients with biological characteristics that clearly differ from CMML without such features, and might have a clinical course closer to that of MDS-RS/*SF3B1* and better than classical CMML. This is currently of special interest given that a lower monocyte count cut-off has been established for the diagnosis of CMML in the new 2022 WHO and ICC classifications, and thus, more patients who would have previously been diagnosed with MDS or MDS/MPN-Unclassifiable (MDS/MPN-U) are currently considered CMML.

## Materials and methods

For this purpose, we included patients with CMML and MDS-RS/*SF3B1* from the Spanish Registry of MDS, the Düsseldorf Registry and the Humanitas Research Hospital in Milan. We considered the 2022 WHO classification criteria for the diagnosis of CMML and MDS-RS/*SF3B1*. Among the CMML patients, we checked for the presence of RS (and *SF3B1*, if applicable, following the same criteria applied for the diagnosis of MDS-RS/*SF3B1*). Accordingly, CMML cases were divided into CMML and CMML-RS/*SF3B1*. Molecular data were collected when available. First, the presence of *SF3B1* mutations, analyzed by either Sanger sequencing (exons 13-15) or next generation sequencing (NGS) (all coding exons), was recorded. In addition, the mutational profile across myeloid-related genes was collected in cases with available NGS data. The clinical characteristics as well as risk stratification considering the CMML-Prognostic Score System (CPSS) in both CMML and CMML-RS/*SF3B1* and the IPSS-R in all three groups were recorded and compared. The OS and cumulative incidence of progression (CIP) to AML were also assessed. OS was calculated from the date of diagnosis to the date of death or last follow-up. The CIP to AML was measured from the date of diagnosis to the date of progression to AML, considering any death not due to progression as a competing event. Similarly, the two groups (CMML and MDS) harboring *SF3B1* mutation regardless of the percentage of RS were also compared.

### Statistical analysis

Baseline characteristics were described as median and range for quantitative variables and frequency and percentage for categorical variables. Comparisons of proportions, medians of variables between the groups were performed with the chi-square test, or Fisher’s exact and median test, as appropriate. OS was calculated with the Kaplan-Meier method, with the log-rank test for comparisons, and CIP to AML was estimated using cumulative incidence functions by competing risks analysis. Groups were compared by Gray’s test. Statistical analyses were performed with SPSS v.24 and R v.4.2.0 software.

## Results

A total of 815 patients were included in the study (CMML, n=319, CMML-RS/*SF3B1*, n=172 and MDS-RS/*SF3B1*, n=324). The main clinical characteristics of the three groups are summarized in **Table 1**.

**Table 1:** Main clinical characteristic of the three groups of patients.

			CMML-RS/*SF3B1* (n=172)	CMML (n=319)	MDS-RS/*SF3B1* (n=324)	CMML-RS/*SF3B1* Vs CMML p	CMML-RS/*SF3B1* vs. MDS-RS/*SF3B1* P
**Male, n (%)**			118 (68.6)	232 (72.7)	192 (59.3)	0.335	**0.041**
**Age, median (range)**			76 (32–94)	75 (28—95)	73 (24-96)	0.318	**0.011**
**Hemoglobin, median (range)**			9.8 (6.2-15.3)	12 (6.6-17.1)	9.750 (3.9-13.4)	**<0.001**	0.844
**Platelets, median (range)**			225 (15-853)	120 (7-1067)	225 (13-443)	**<0.001**	0.896
**Leukocyte count, median (range)**			5.6 (1.7-48)	8.5 (2.5-111.7)	5.04 (0.5-12.69)	**<0.001**	**0.024**
**Monocyte count, median (range)**			0.791 (0.5-22.419)	1.9 (0.5-43.8)	0.32444 (0-0.984)	**<0.001**	**<0.001**
**Monocyte %, median (range)**			14.2929 (10-51.08)	23.1729 (10-66.97)	6.6051 (0-23)	**<0.001**	**<0.001**
**Monocyte count** **x10^9^/L**		**<0.5**	0	0	256 (79)	**<0.001**	**<0.001**
		**0.5-0.9**	125 (72.7)	14 (4.4)	68 (21)		
		**≥1**	47 (27.3)	305 (95.6)	0		
**Neutrophils, median (range)**			2.6 (0.003-12.593)	4.17935 (0.1-70.4)	2.95 (0.036-9.720)	**<0.001**	**0.025**
**Peripheral blood blasts, median (range)**			0 (0-4)	0 (0-10)	0 (0-1)	**0.007**	0.068
**Bone marrow blasts, median (range)**			2 (0-18)	3 (0-19.4)	1 (0-4)	**<0.001**	0.038
**FAB CMML n (%)**		**MD**	165 (95.9)	232 (72.7)	–	**<0.001**	–
		**MP**	7 (4.1)	87 (27.3)			
**WHO 2022, CMML n (%)**		**CMML-1**	166 (97)	278 (87.1)	–	**0.001**	–
		**CMML-2**	6 (3)	41 (12.9)			
**Bone marrow cellularity** **n (%)**		**Hypocellular**	1/156 (0.6)	5/278 (1.8)	8/294 (2.7)	0.296	0.324
		**Normal**	39/156 (25)	55/278 (19.8)	73/294 (24.8)		
		**Hypercellular**	116/156 (74.4)	218/278 (78.4)	213/294 (72.4)		
		**1**	46/163 (28.2)	88/305 (28.9)	83/278 (29.9)		
		**2**	55/163 (33.7)	124/305 (40.7)	87/278 (31.3)		
		**3**	62/163 (38)	80/305 (26.2)	108/278 (38.8)		
**RS, n (%)**		**Median, range**	42 (6-99)	0 (0-13)	42 (5-98)	**<0.001**	0.838
		**<5%**	0	311 (97.5)	0	**<0.001**	0.133
		**5%-14%**	3 (1.7)	8 (2.5)	14 (4.3)		
		**≥15%**	169 (98.3)	0	310 (95.7)		
***SF3B1* mutation, n (%)**			61/87 (70.1)	0/93	98/124 (79)	**<0.001**	0.139
**Dyserythropoiesis., n (%)**			145/149 (97.3)	172/280 (61.4)	255/259 (98.5)	**<0.001**	0.471
**Dysgranulopoiesis, n (%)**			96/141 (68.1)	204/241 (84.6)	167/233 (71.7)	**<0.001**	0.462
**Dysmegakaryopoiesis, n (%)**			79/142 (55.6)	152/260 (58.5)	144/240 (60)	0.584	0.403
**CPSS** **n (%)**		**Low**	66 (38.4)	180 (56.4)	–	**0.001**	–
		**Int-1**	74 (43)	87 (27.3)			
		**Int-2**	28 (16.3)	41 (12.9)			
		**High**	4 (2.3)	11 (3.4)			
**IPSS-R** **n (%)**		**Very Low**	57/170 (33.5)	84/292 (28.8)	116 (35.8)	0.189	**0.016**
		**Low**	75/170 (44.1)	131/292 (44.9)	164 (50.6)		
		**Intermediate**	29/170 (17.1)	43/292 (14.7)	36 (11.1)		
		**High**	8/170 (4.7)	27/292 (9.2)	3 (0.9)		
		**Very high**	1/170 (0.6)	7/292 (2.4)	5 (1.5)		
**Risk (IPSS-R)** **n (%)**		**Low**	149/172 (86.6)	231/292 (79.1)	292 (90.1)	**0.042**	0.238
		**High**	23/172 (13.4)	61/292 (20.9)	32 (9.9)		

CMML, chronic myelomonocytic leukemia; RS, ring sideroblasts; MDS, myelodysplastic syndrome; FAB, French-American-British; MD, myelodysplastic; MP, myeloproliferative; WHO, World Health organization; CPSS, Chronic myelomonocytic leukemia Prognostic Score System; IPSS-R, Revised International Prognostic Score System.Significant differences are highlighted in bold.

Within the group of CMML-RS/*SF3B1*, 165 (95.9%) had the myelodysplastic phenotype according to the French-American-British (FAB) classification and 166 (96.5%) had a low percentage of blasts (CMML1 according to WHO 2022). It is of note that the percentage of RS was ≥15% in most patients (169, 98.3%) with a median of 42 (6-99%), and *SF3B1* mutation was present in 61 out of 87 evaluable patients (70.1%). The median hemoglobin level was 9.8 (range 6.2-15.3). In most CMML-RS/*SF3B1* (125, 72.7%) the monocyte count in peripheral blood was between 0.5 and 0.9 x10^9^/L (previously referred to as oligomonocytic CMML) and low risk categories by CPSS were predominant (low/intermediate-1 140, 81.4%), also after stratifying patients as low and high risk by IPSS-R considering the cut-off of 3.5 (n=149, 86.6%) (**Table 1**). Around 70% of CMML cases had MD subtype and almost 90% CMML-1 in this series.

CMML-RS/*SF3B1* significantly differed from classical CMML in the main clinical characteristics of the patients except for age, gender, bone marrow cellularity, dysmegakaryopoiesis and risk by IPSS-R, whereas it shared most of the clinical features with MDS-RS/*SF3B1*, excluding total and subsets of white blood cell counts. As expected, the median total monocyte count was higher in CMML-RS-*SF3B1* compared to MDS-RS/*SF3B1*, but this value was still less than 1 x10^9^/L (125, 72.7%) in most patients.

*SF3B1* mutation was present in 61 patients with CMML and 98 patients with MDS. Considering this molecular feature, CMML-*SF3B1* and MDS-*SF3B1* were phenotypically very close, barely differing in absolute and relative monocyte count. Again, the absolute monocyte count was lower than 0.5 x10^9^/L in most MDS-*SF3B1* patients, whereas in the group of CMML-*SF3B1* a range between 0.5 and 1 x10^9^/L was predominant (**Table 2**).

**Table 2:** Main clinical characteristic of the two groups of patients with *SF3B1* mutation.

		CMML-*SF3B1* (n=61)	MDS-*SF3B1* (n=98)	P
**Male, n (%)**		36 (59)	63 (64.3)	0.505
**Age, median (range)**		77 (48-94)	74 (41-96)	0.146
**Hemoglobin, median (range)**		10 (6.8-15.3)	9.850 (6.6-13.2)	0.581
**Platelets, median (range)**		246 (23-853)	241 (26-437)	0.821
**Leukocyte count, median (range)**		5.6 (3.3-22.8)	5.6 (1.73-11.94)	0.980
**Monocyte count, median (range)**		0.8 (0.5-5.470)	0.4 (0.035-0.984)	**<0.001**
**Monocyte %, median (range)**		14.4578 (10-37.22)	7.6655 (1-17)	**<0.001**
**Monocyte count** **x10^9^/L**	**<0.5**	0	72 (73.5)	**<0.001**
	**0.5-1**	48 (78.7)	26 (26.5)	
	**≥1**	13 (21.3)	0	
**Neutrophils, median (range)**		2.6895 (0.003-9.58)	3.30400 (0.265-8.177)	0.075
**Bone marrow blasts, median (range)**		1 (0-10)	1 (0-4)	0.070
**Bone marrow cellularity** **n (%)**	**Hypocellular**	0	2/82 (2.4)	0.441
	**Normal**	14/51 (27.5)	26/82 (31.7)	
	**Hypercellular**	37/51 (72.5)	54/82 (65.9)	
**Dysplastic lines**	**1**	24 (39.3)	29/91 (31.9)	0.632
	**2**	15 (24.6)	26/91 (28.6)	
	**3**	22 (36.1)	36/91 (39.6)	
**RS, n (%)**	**Median, range**	45 (6-91)	41 (5-94)	0.498
	**5%-14%**	3 (4.9)	14 (14.3)	0.063
	**≥15%**	58 (95.1)	84 (85.7)	
**Dyserythropoiesis., n (%)**		57/58 (98.3)	85/87 (97.7)	1.000
**Dysgranulopoiesis, n (%)**		32/58 (55.2)	55/83 (66.3)	0.182
**Dysmegakaryopoiesis, n (%)**		23/58 (39.7)	44/85 (51.8)	0.154
**CPSS** **n (%)**	**Low**	29 (47.5)	–	–
	**Intermediate-1**	26 (42.6)		
	**Intermediate-2**	6 (9.8)		
	**High**	0		
**IPSS-R** **n (%)**	**Very Low**	28 (45.9)	40 (40.8)	0.347
	**Low**	24 (39.3)	49 (50)	
	**Intermediate**	8 (13.1)	9 (9.2)	
	**High**	1 (1.6)	0	
	**Very high**	0	0	
**Risk (IPSS-R)** **n (%)**	**Low**	58 (95.1)	92 (93.9)	1.000
	**High**	3 (4.9)	6 (6.1)	

CMML, chronic myelomonocytic leukemia; RS, ring sideroblasts; MDS, myelodysplastic syndrome; CPSS, CMML-Prognostic Score System; IPSS-R, Revised International Prognostic Score System.Significant differences are highlighted in bold.

**Figure 1:**
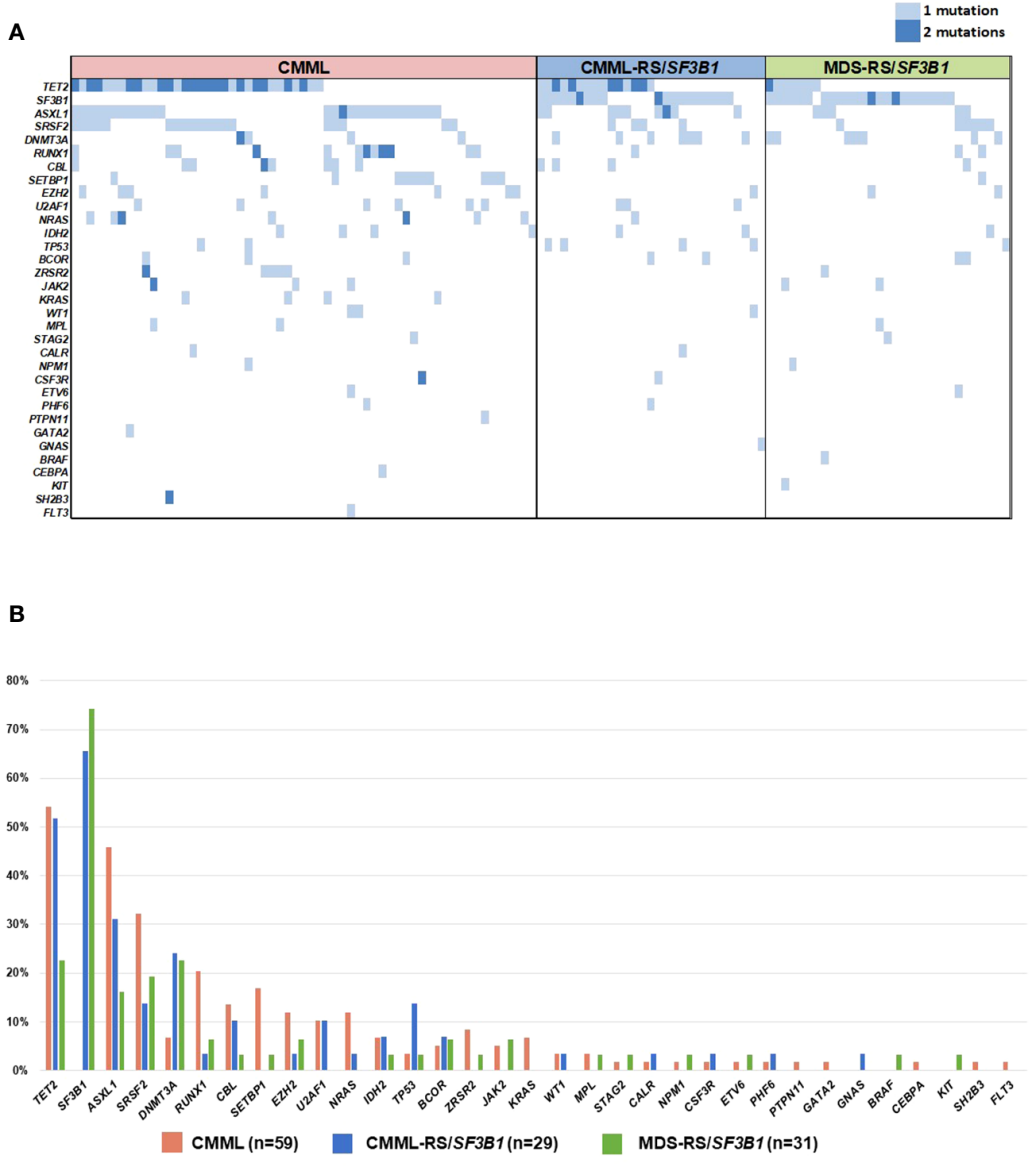
**(A)** Molecular profile of the three groups of patients. **(B)** Frequency of gene mutations in the three groups of patients.

Data from NGS was available in 29, 61 and 32 patients with CMML-RS/*SF3B1*, CMML and MDS-RS/*SF3B1*, respectively. The molecular profiles of the three groups (CMML, CMML-RS/*SF3B1* and MDS-RS/*SF3B1*), as well as the percentage of mutations, can be seen in **Figures 1A, B**. Briefly, the most frequent mutations were *TET2* (mostly multi-hit), *ASXL1* and *SRSF2* in CMML, *TET2* (mostly multi-hit), *SF3B1* and *ASXL1* in CMML-RS/*SF3B1*, and *SF3B1*, *DNMT3A* and *TET2* in MDS-RS/*SF3B1*. As expected, other splicing mutations (*SRSF2*, *U2AF1*, *ZRSR2*) were exceptionally found (**Figures 1A, B**) in *SF3B1* mutated patients.

**Figure 2:**
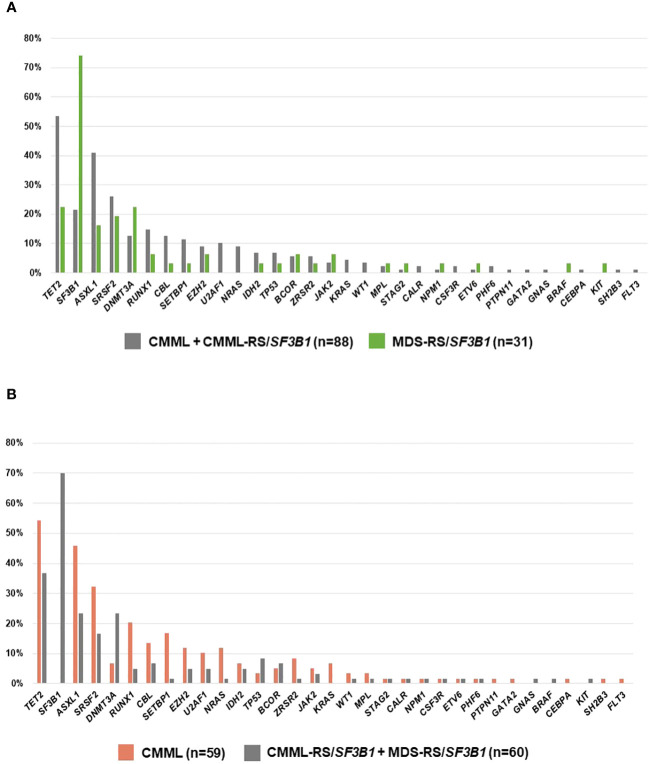
**(A)** Frequency of gene mutations in CMML (with and without ring sideroblasts (RS)/*SF3B1*) vs. MDS-RS/*SF3B1.* **(B)** Frequency of mutations in CMML vs. the two groups with ring sideroblasts (RS)/*SF3B1*.

Then, we compared the molecular landscape across groups defined by phenotype. On one hand, when we compared MDS-RS/*SF3B1* vs. CMML (CMML+CMML-RS/*SF3B1*), the latter showed a significantly higher frequency of mutations in *TET2* (p=0.003), mostly multi-hit, and in *ASXL1* (p=0.013), and fewer in *SF3B1* (p<0.001) (**Figure 2A**). On the other hand, when we compared CMML vs. RS/*SF3B1* patients (CMML-RS/*SF3B1* + MDS-RS/*SF3B1*), CMML was enriched in mutations in *TET2* (p=0, 054), *ASXL1* (p=0.010), *SRSF2* (p=0.048), *RUNX1* (p=0.011), *SETBP1* (p=0.004) and *NRAS* (p=0.026) but lacked mutations in *DNMT3A* (p=0.012) and, obviously (by definition), *SF3B1* (**Figure 2B**). In addition, the CMML-RS/*SF3B1* group was also compared individually with CMML and MDS-RS/*SF3B1* (**Table 3**).

**Table 3:** Comparison of similar studies performed in patients with MDS or MDS/MPN and the present series.

	MANGAONKAR^13^		WUDHIKARN^12^		XICOY/Present series	
	n=778		n=859		n=815	
	*SF3B1* ^MUT^MDS/MPN_vs_		CMMLSF3B1^MUT^vs		CMML with RS/*SF3B1* vs	
	*SF3B1* ^WT^MDS/MPN	*SF3B1* mut MDS	CMML *SF3B1* ^WT^	*SF3B1* ^MUT^ MDS-RS	CMML without RS/*SF3B1*	MDS with RS/SF3B1
CLINICAL DIFFERENCES					Hemoglobin	
					Platelet	
	Sex				WBC	
	Hemoglobin		Hemoglobin		AMC and %	
	WBC	Sex	WBC	Leukocyte	ANC	Age
	ANC	WBC ANO	ANC	Monocyte count Platelet count	PB blasts	Gender
	AMC	AMC	AMC	IMCs	BM blasts	WBC
	Platelet	Platelet	Platelet count	PB blasts	FAB subtype	AMC and%
	BM RS	PE blats	RS	BM blasts	Dysplastic lines	IPSS-R
	PB blats	BM blasts	FAB subtype		RS (median, %)	
	BM blasts		Cytogenetic risk		Dyserythropoieseis	
	Abnormal karyotype				Dysgranulopiesis	
					CPSS	
					IPSS-R (low/High)	
MOLECULAR DIFFERENCES	-	*JAK2 V617F* (enriched in *SF3B1* ^MUT^ MDS/MPN)	*ASXL1* and *SRSF2* (enriched in CMML *SF3B1* ^WT^	*RUNX1* (enriched in CMML SF3B1^WT^)	*DNMT3A*, *SF3B1* (enriched in CMML-RS/*SF3B1 RUNX1*, *SETBP1* (enriched in CMML without RS/*SF3B1*	TET2 (enriched in CMML-RS/*SF3B1*)
AML-T	Lower	NS	Lower	Higher	Lower	NS
os	Higher	NS	NS (trend)	NS (trend)	Higher	NS

MDS/MPN, myelodysplastic syndrome/myeloproliferative neoplasm; CMML, chronic myelomonocytic leukemia; MDS, Myelodysplastic syndrome; RS, ring sideroblasts; WBC, leukocyte count; ANC, absolute neutrophil count; AMC, absolute monocyte count; BM, bone marrow; RS, ring sideroblasts; PB, peripheral blood; FAB, French-American-British; IMC, immature myeloid cells; CPSS, CMML-Prognostic Score System; IPSS-R, Revised International Prognostic Score; AML-T, acute myeloid leukemia transformation; OS, overall survival.

**Figure 3:**
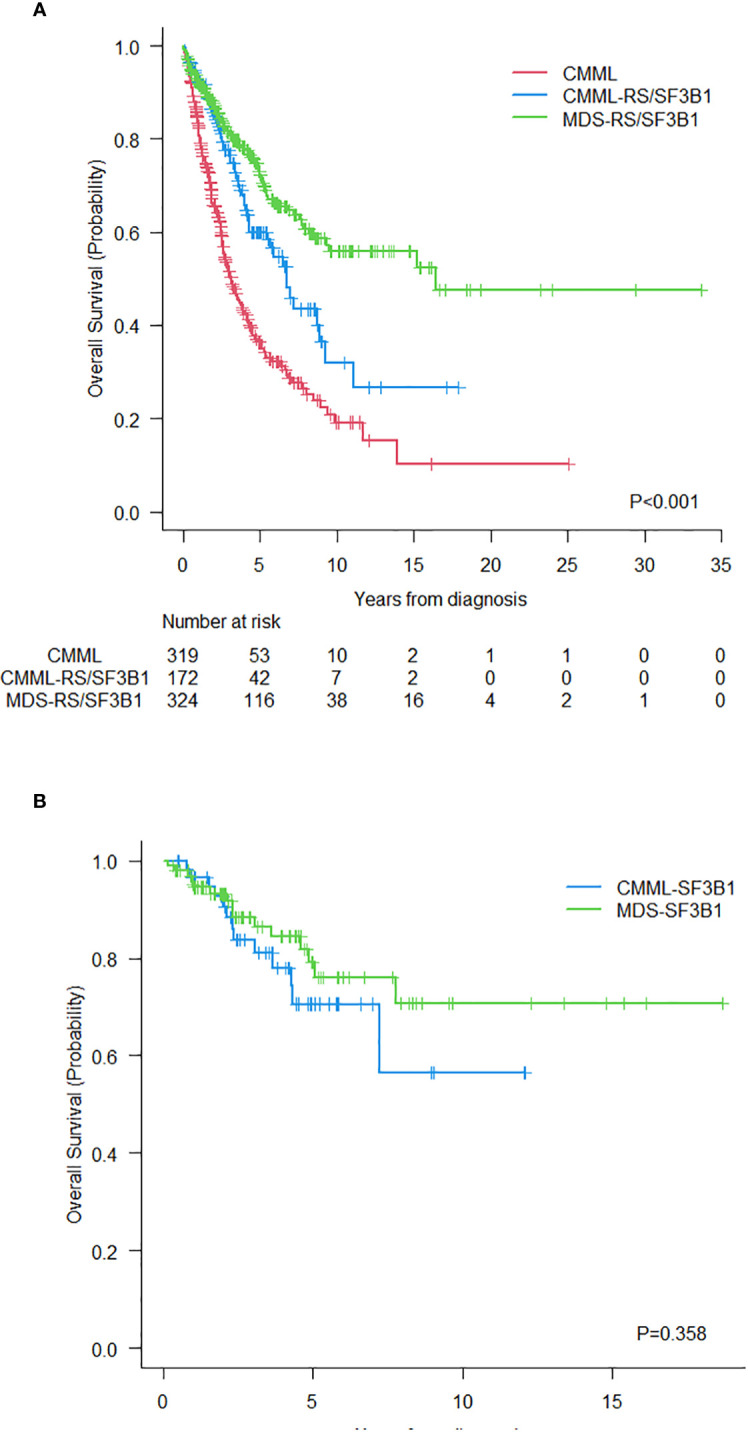
**(A)** Overall survival of the three groups of patients. **(B)** Overall survival of patients with CMML and MDS with *SF3B1* mutation.

The median follow-up for alive patients in the whole series was 3.25 (0.26-33.66) years: 2.96 (0.35-17.9) for CMML-RS/*SF3B1*, 2.51 (0.33-25.1) for CMML and 3.96 (0.26-33.66) for MDS-RS/*SF3B1*. The median OS was 6.38 years (95%CI 5.2-7.49). Differences in OS among the three groups were statistically significant: 6.75 years (95% CI 5.41-8.09) for CMML-RS/*SF3B1* vs. 3.17 years (95% CI 2.56-3.79) for CMML vs. 16.47 years (NA) for MDS-RS/*SF3B1*, p<0.001 (**Figure 3A**). When comparing OS between CMML-RS/*SF3B1* and both CMML and MDS-RS/*SF3B1, the* differences remained significant (p<0.001 and p=0.008). Regarding patients with *SF3B1* mutation, the survival was not significantly different. At 8 years, the OS was 57% (95%CI 30%-84%) in CMML with *SF3B1* mutation and 71% (95%CI 56%-86%) in MDS with *SF3B1* mutation (**Figure 3B**).

**Figure 4:**
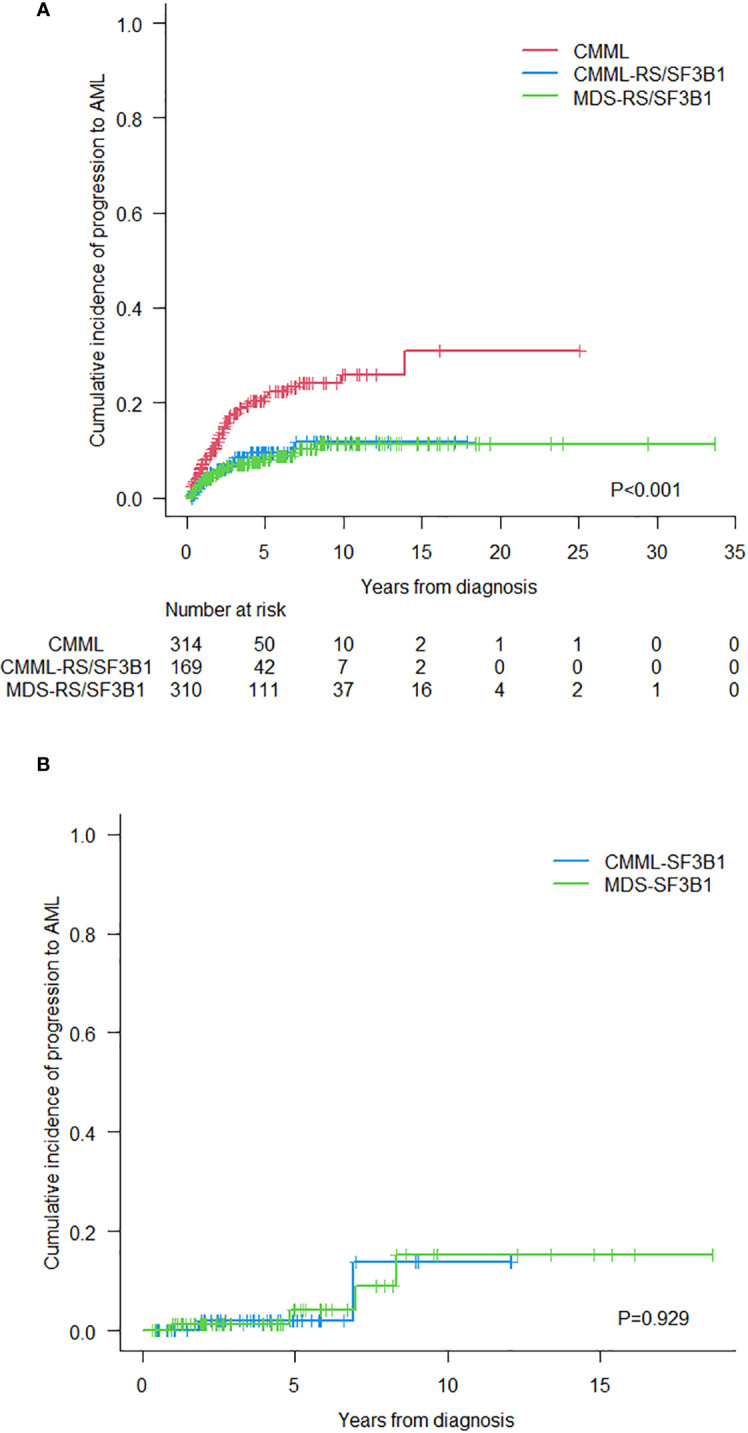
**(A)** Cumulative incidence of progression to acute myeloid leukemia in the three groups of patients. **(B)** Cumulative incidence of progression to acute myeloid leukemia in CMML and MDS with *SF3B1* mutation.

The 8-year CIP to AML transformation (95%CI) in the 3 groups (CMML-RS/*SF3B1*, CMML and MDS-RS/*SF3B1*) was: 12% (6%-19%), 24% (19%-30%) and 10% (7%-15%), respectively. This outcome did not significantly differ between the groups with *SF3B1* mutation: for CMML-*SF3B1* 14% (1%-43%) and for MDS*-SF3B1* 9% (2%-24%) (p=0.929) (**Figures 4A, B**).

Finally, we focused on the group of MDS-RS/*SF3B1* looking for a possible influence of absolute monocyte count in peripheral blood on outcomes. There was no correlation between the percentage of RS and monocyte count (p=0.120 but we observed that the greater the number of monocytes (<0.2, 0.3-0.4 and ≥0.5), the worse the survival (8-year OS, 95% CI: 78% (63%-87%) vs. 60% (50%-69%), vs. 41% (22%-58%), p=0.010) (**Supplementary Figure 1A**). Within the group with *SF3B1* mutation, a better OS was observed in the presence of a very low monocyte count (<0.2 vs. ≥0.2) (8-yr OS 89% (43%-98%) vs. 69% (50%-82%), p=0.088) (**Supplementary Figure 1B**).

## Discussion

CMML was first described by the FAB group in 1976 as a chronic disease in which the monocyte count was higher than 1 x10^9^/L and the monocytes often showed atypical morphological features (16). The presence of RS was initially reported as a frequent feature of MDS (at that time called sideroblastic anemia) in the 1982 FAB classification (17). Approximately 30%-35% of MDS patients present with RS, erythroblasts in the bone marrow with at least five iron granules that cover one-third or more of the perinuclear region, corresponding to mitochondrial ferritin (18, 19). In FAB and previous WHO classification systems for myeloid neoplasms, cases presenting with both an absolute and relative monocyte count in peripheral blood > 1×10^9^/L and ≥10% of the leukocyte count were considered as CMML regardless of other morphological findings, such as the presence of RS in the bone marrow. However, the greater preeminence given to the monocyte count in peripheral blood over the proportion of RS in the bone marrow was not evidence-based.

Molecular techniques, and especially NGS, have improved the knowledge of the biology of most myeloid neoplasms. It is well known that more than 90% of patients with CMML have somatic mutations, with splicing factor mutations such as *SRSF2*, *U2AF1*, *ZRSR2* and *SF3B1* being highly prevalent, especially *SRSF2* (20–24). Because *SRSF2* has not been detected in the healthy elderly population (unlike *TET2* and *ASXL1*) and although this mutation is not specific of this disease, it is useful as a clonal marker in the differential diagnosis of other causes of monocytosis (25). Moreover, co-occurrence of mutations in *SRSF2* and *TET2*, and the presence of multiple *TET2* mutations, are considered hallmarks of CMML (21, 22, 26). In MDS, *SF3B1* mutations are present in 80% of patients with MDS with RS (90% of MDS-RS-UD and in 70% MDS-RS-MD based on the 2017 WHO classification)^10^. According to the 2017 WHO classification, its presence allows the diagnosis of MDS with RS when the percentage of RS ranges between 5%-14%, whereas its detection can be ignored when this proportion is greater than or equal to 15%. This subtype of MDS is characterized by a good prognosis in terms of OS and CIP to AML and by responses to Luspatercept (6–8). More recently, *SF3B1* mutation was incorporated as a defining genetic feature, named MDS with low blasts and *SF3B1* mutation by the 2022 WHO classification, and MDS with mutated *SF3B1* by the ICC of the same year (2, 3). In patients with CMML, mutations in *SF3B1* have also been associated with lower hemoglobin levels with no impact in prognosis (9, 21).

The Spanish Group of MDS suggested that the proportion of RS in bone marrow could be a much more powerful prognostic indicator than the absolute monocyte count in peripheral blood in CMML (27). Two recent studies have addressed this issue focusing on the role of *SF3B1* mutation in the phenotype and clinical course of MDS/MPN neoplasms. The study by Wudhikam et al. compared CMML-*SF3B1^MUT^* to CMML-*SF3B1* ^WT^ and MDS-*SF3B1* ^MUT^, while Mangaonka^r^ et al. focused on the distinction between MDS/MPN-*SF3B1^MUT^*, its wild-type counterparts, and the group of MDS-*SF3B1^MUT^* (12, 13). In both studies, the CMML phenotype was closer to that of MDS as long as they shared the *SF3B1* mutation. Moreover, no significant differences were found in terms of OS and the transformation to AML between MDS/MPN-*SF3B1* ^MUT^ and MDS-*SF3B1^MUT^* in the study by Mangaonkar et al., whereas only leukemia-free survival of CMML-*SF3B1^MUT^* was poorer than that of MDS-*SF3B1* ^MUT^ in the series of Wudhikarn et al. (**Table 3**) (12, 13).

Unlike the study of Wudhikarn et al., we adopted the 2022 WHO classification in our series and, accordingly, patients with a monocyte count between 0.5 and 0.9 x10^9^/L and ≥10% were considered as CMML (with or without RS/*SF3B1*) (12). Indeed, the majority of CMML-RS/*SF3B1* patients in our series fulfilled this criterion (the so-called oligomonocytic CMML) and in the past, these patients would have been classified as MDS or MDS/MPN-U following the criteria of previous WHO classifications (28). In other words, the presence of RS or *SF3B1* mutation in CMML is associated with a low absolute monocyte count in peripheral blood and, based on our findings, only the relative monocyte count allocates a patient into the CMML category. Several studies have suggested that oligomonocytic CMML shares many characteristics with classical CMML and may be considered as an early stage with better prognosis (28–32).

Regarding other clinical features, we observed that when RS are predominant and regardless of the presence of *SF3B1* mutation, CMML and MDS patients share the main clinical characteristics, supporting the hypothesis that RS play a greater role than the monocyte count in determining the phenotype, which is closer to MDS than CMML. In line with these findings, the molecular landscape of CMML-RS/*SF3B1* was closer to that of MDS-RS/*SF3B1* than to CMML (without RS/*SF3B1*), being enriched in *DNMT3A* mutations, and with a lower frequency of mutations in *SRSF2*, *RUNX1* and *SETBP1* (**Table 3**). Furthermore, when we grouped together and compared patients according to their main defining morphological findings (monocytosis or RS/*SF3B1*, **Figures 2A, B**), the most notable differences were observed between CMML and patients with RS/*SF3B1* (both MDS and CMML). Even though numbers of patients with available NGS in the three series are small, it is of some interest the observation that *TP53* mutation- – which is generally reported in <5% of CMML patients-, is in the 13%-14% range in CMML-RS/*SF3B1* (possibly due to some cases of CMML-RS with unknown *SF3B1* status) (**Figures 1A, B**).

Nevertheless, the OS of CMML-RS/*SF3B1* was not as good as MDS-RS/*SF3B1* and this finding cannot be attributed to a higher CIP to AML. Patients with CMML-RS/*SF3B1* were older, and this is usually associated with the presence of more comorbidities, but the causes of death different from AML transformation did not apparently differ (data not shown). Based on our results and published data, a prospective study is required to better clarify the impact of RS/*SF3B1* on OS and AML transformation (12, 13). The frequency of poor prognosis mutations, as defined in CPSS molecular scoring system, such as *ASXL1*, *SETBP1*, *RUNX1* and *NRAS*, was higher in CMML than in CMML-RS/*SF3B1*, which may explain the poorer prognosis of the former group. In contrast, the presence of multi-hit mutations in *TET2* was similar in CMML cases (with and without RS/*SF3B1*) and more frequent than in the MDS-RS/*SF3B1* group, which explains the higher monocyte count that characterizes CMML, while cases with RS/*SF3B1* (CMML or MDS) frequently showed co-mutations in *DNMT3A*, as previously described (29, 33). Unfortunately, molecular data were insufficient to evaluate whether mutations defined in the IPSS-M as having poor prognosis in patients with MDS-*SF3B1* have the same impact in cases with CMML-*SF3B1* (34).

In our cohort of MDS-RS/*SF3B1* mutation the absolute monocyte count in peripheral blood negatively influenced OS. These data are in line with those of Kasprzak et al. and Silzle et al., which, in a population of MDS with low blast count, have recently shown that monocytosis >0.6 x10^9^/L is associated with a shorter OS, also in the subtype enriched with RS. This suggests that MDS with monocytosis and CMML with RS may be located in a “gray zone” that makes diagnosis challenging but, on the other hand, supports a therapeutically similar approach in these cases (14, 15). Altogether, this study supports the notion that classifications based on selected clinical characteristics, generally with arbitrarily chosen thresholds, have always inherent limitations. The results of this study raise the question whether patients with CMML-RS/*SF3B1* and monocytosis <1.0 x10^9^/L should be properly classified as CMML or should be rather classified as MDS with RS/*SF3B1* mutation and relative monocytosis similar to what we observe in myelofibrosis when it presents with monocytosis (35). Furthermore, for patients with absolute monocyte count in the range 0.5-0.9 x 10^9^/L to distinguish the entities MDS-RS/*SF3B1* or CMML-RS/*SF3B1* on the base of relative monocytosis (i.e. <10% vs ≥10%) is biologically irrational. Some studies have shown that oligomonocytic CMML patients resemble CMML mainly for their immunophenotypic and genomic features, and thus, absolute monocyte count has been lowered from 1 to 0.5 x10^9^/L in the 2022 WHO/ICC classifications, provided that an acquired cytogenetic or molecular clonality is found. The results reported in this manuscript, however, indicate that in such subset of patients, the diagnostic criteria should at least include the exclusion of patients with RS/*SF3B1*.

This is a registry-based study and, therefore, includes retrospective data and has its inherent limitations; First, we cannot rule out a selection bias in the inclusion of patients, especially with the CMML-RS/*SF3B1* phenotype and thus, we could not determine the real prevalence of this subtype of CMML; Second, a centralized diagnosis was missing and we were not able to exhaustively revise causes of death; third, the use of Sanger and NGS, with different sensitivity for analyzing *SF3B1*, may bias the results; Finally, the availability of a complete molecular profile was restricted to a few patients and also, the type of variants and variant allele frequency were missing in some patients, limiting the evaluation of the influence of existing mutations on the phenotype and evolution of the cases.

In conclusion, CMML harboring RS/*SF3B1* mutation resembles MDS-RS/*SF3B1* in terms of phenotype and prognosis and clearly differs from classical CMML. The presence of ≥15% RS and/or *SF3B1* mutation in CMML is associated with a low monocyte count and *SF3B1* mutation clearly improves the prognosis of CMML.

## Data availability statement

This study has been carried out collecting data from different registries. Requests to access these datasets should be directed to BX, bxicoy@iconcologia.net.

## Ethics statement

The studies involving humans were approved by Comitè d’etica d’Investigació de l’Hospital Universitari Germans Trias i Pujol. The studies were conducted in accordance with the local legislation and institutional requirements. The participants provided their written informed consent to participate in this study.

## Author contributions

BX: Writing – review & editing, Writing – original draft, Visualization, Validation, Supervision, Software, Resources, Project administration, Methodology, Investigation, Funding acquisition, Formal analysis, Data curation, Conceptualization. HP: Writing – review & editing, Data curation. MM: Writing – review & editing, Methodology, Formal analysis. UG: Writing – review & editing, Data curation. MA: Writing – review & editing, Data curation. MT: Writing – review & editing, Data curation. LP: Writing – review & editing, Data curation. EO: Writing – review & editing, Data curation. MD: Writing – review & editing, Data curation. FS: Writing – review & editing, Data curation. MD-B: Writing – review & editing, Data curation. AE: Writing – review & editing, Data curation. AM: Writing – review & editing, Data curation. LL: Writing – review & editing, Data curation. AA: Writing – review & editing, Data curation. MU: Writing – review & editing, Data curation. AP: Writing – review & editing, Data curation. VR: Writing – review & editing, Data curation. FH: Writing – review & editing, Data curation. MD-C: Writing – review & editing, Data curation. LZ: Writing – review & editing, Data curation.

## Supplementary material

Supplementary Figure 1
**(A)** Overall survival of patients with MDS-RS/*SF3B1* mutation according to monocyte count. **(B)** Overall survival of patients with MDS-*SF3B1* mutation according to monocyte count.
