# Supplementary figures and images for: Chronic myelomonocytic leukemia with ring sideroblasts/SF3B1 mutation presents with low monocyte count and resembles myelodysplastic syndromes with-RS/SF3B1 mutation in terms of phenotype and prognosis
Source: Front Oncol. 2024 Jul 1;14:1385987. doi: 10.3389/fonc.2024.1385987 (PMC11246989; doi:10.3389/fonc.2024.1385987)

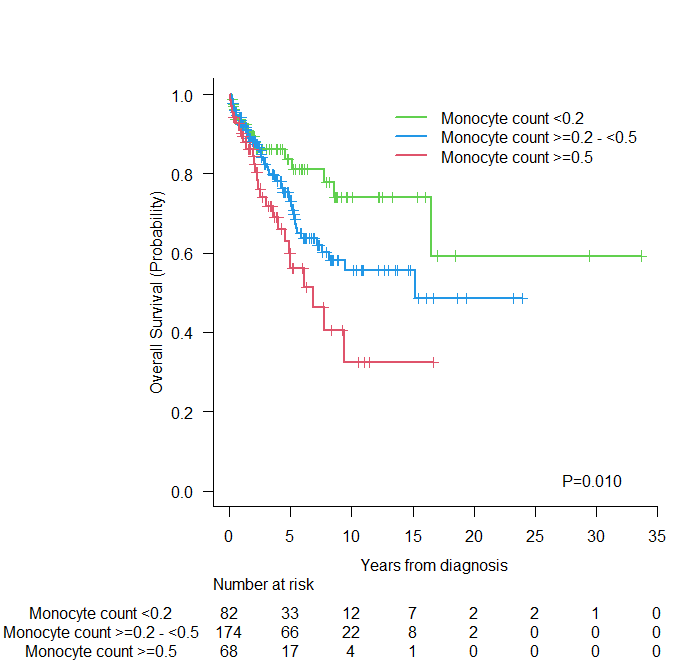

Supplement: Supplementary Figure 1 — (A) Overall survival of patients with MDS-RS/SF3B1 mutation according to monocyte count. (B) Overall survival of patients with MDS-SF3B1 mutation according to monocyte count. [file Image_1.tiff]

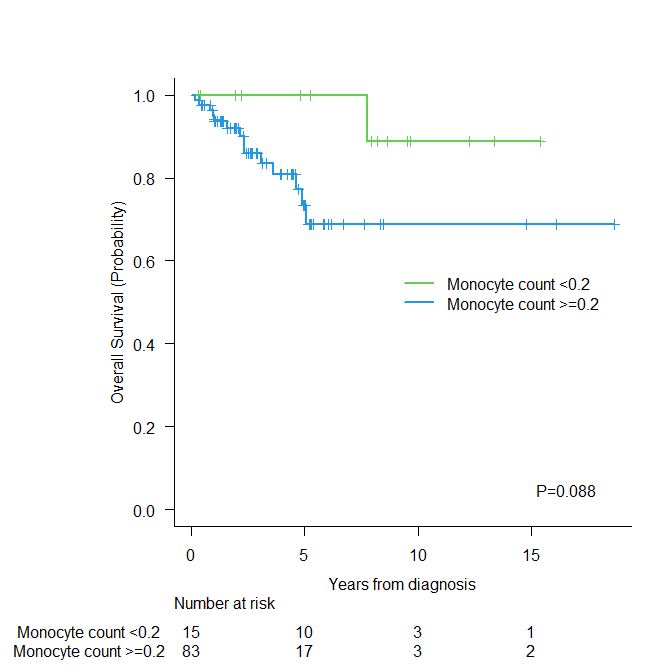

Supplement: Supplementary file 2 [file Image_2.tiff]
